# Supplementary material for: Hemopexin Suppresses Hepatocellular Carcinoma via TNF-α-Mediated Mitochondrial Apoptosis
Source: Cancers (Basel). 2025 Sep 11;17(18):2969. doi: 10.3390/cancers17182969 (PMC12468737; doi:10.3390/cancers17182969)
Supplement: Supplementary file 1 [file cancers-17-02969-s001.zip › cancers-3828311-supplementary.pdf]

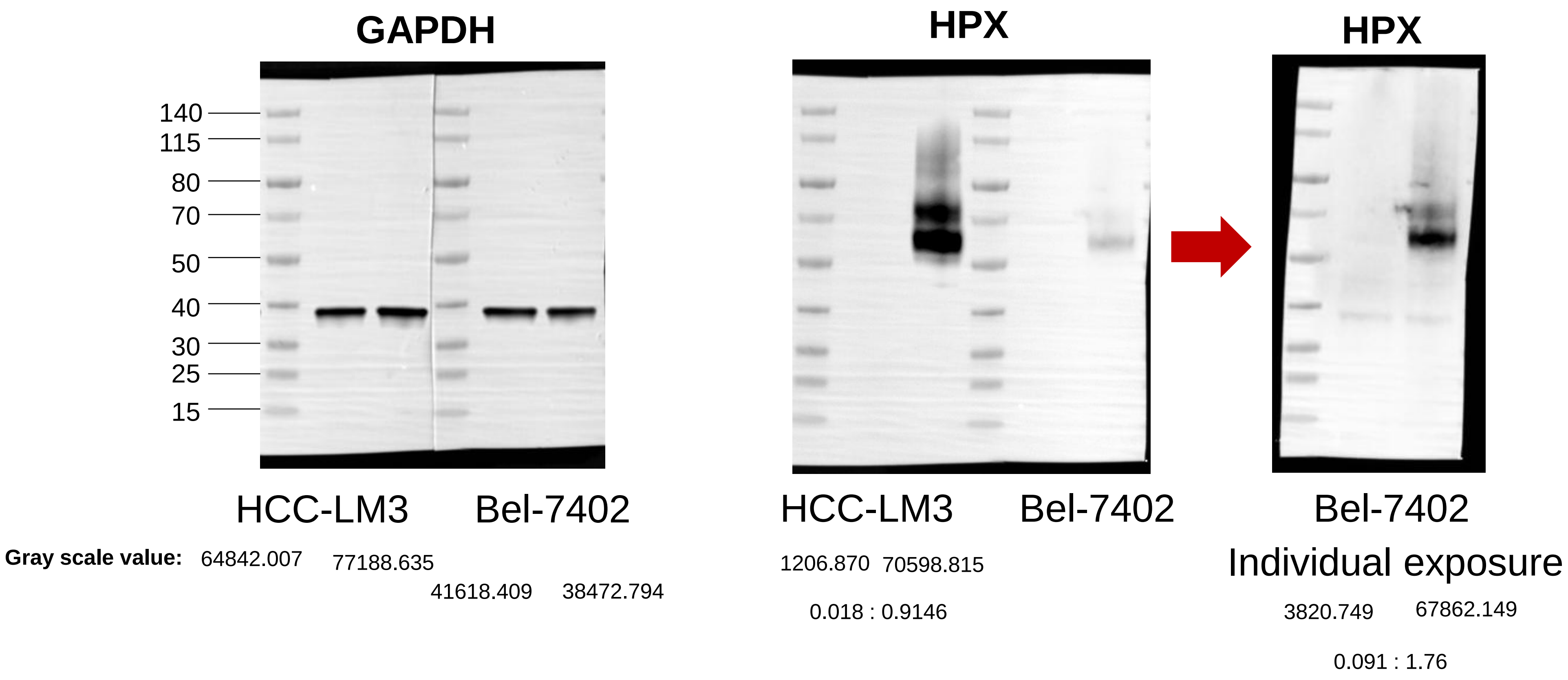
GAPDH
140
115
80
70
50
40
30
25
15
HCC-LM3
Bel-7402
Gray scale value:
64842.007
77188.635
41618.409
38472.794
HPX
HCC-LM3
Bel-7402
1206.870
70598.815
0.018 : 0.9146
HPX
Bel-7402
Individual exposure
3820.749
67862.149
0.091 : 1.76

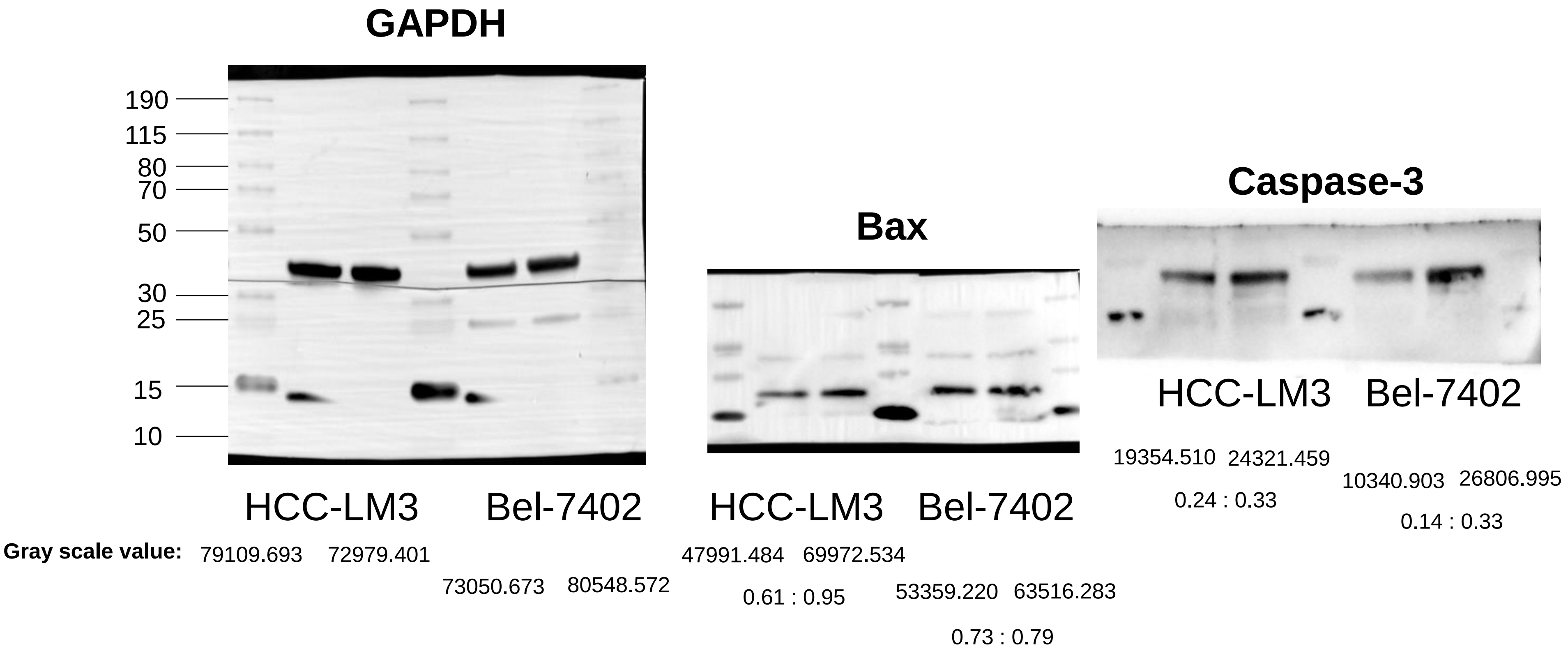
GAPDH
190
115
80
70
50
30
25
15
10
HCC-LM3
Bel-7402
Gray scale value:
79109.693
72979.401
73050.673
80548.572
Bax
HCC-LM3
Bel-7402
47991.484
69972.534
0.61 : 0.95
53359.220
63516.283
0.73 : 0.79
Caspase-3
HCC-LM3
Bel-7402
19354.510
24321.459
0.24 : 0.33
10340.903
26806.995
0.14 : 0.33

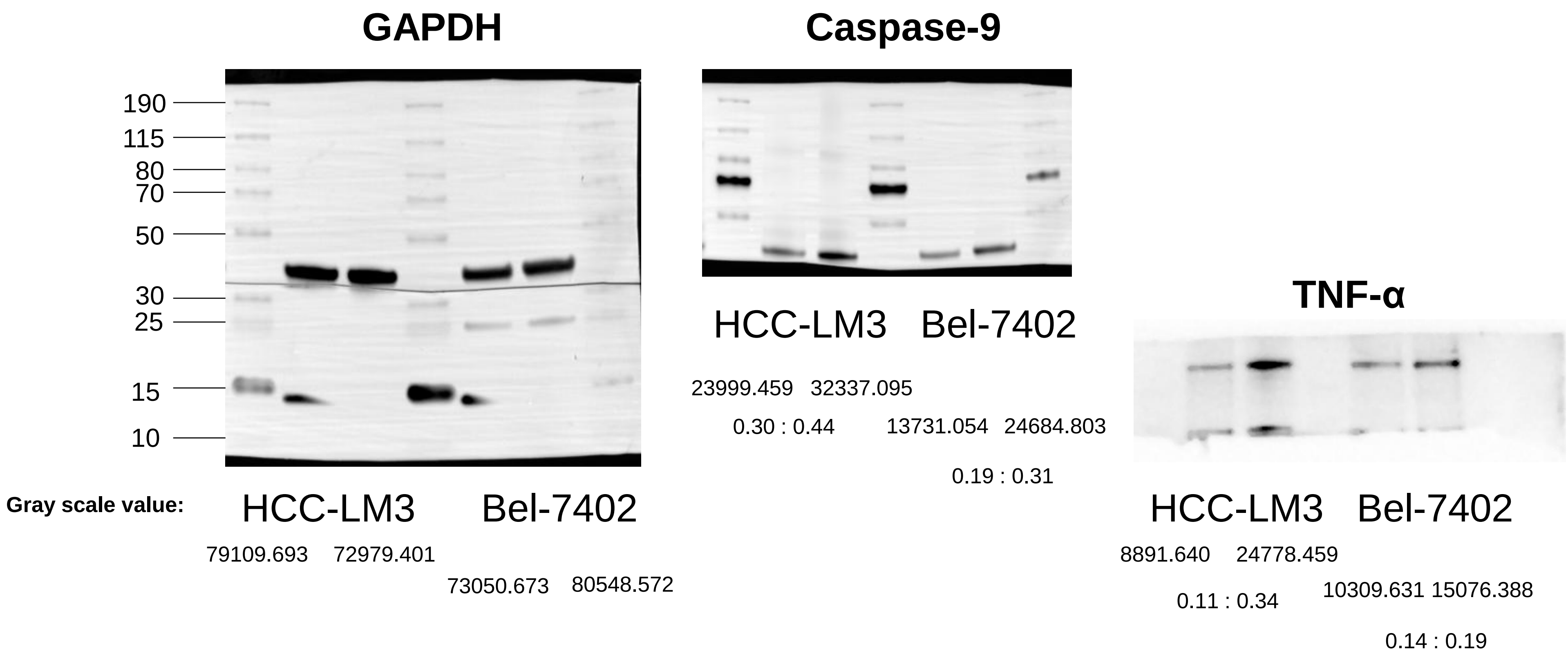
GAPDH
Caspase-9
TNF-α
190
115
80
70
50
30
25
15
10
Gray scale value:
HCC-LM3
Bel-7402
79109.693
72979.401
73050.673
80548.572
HCC-LM3
Bel-7402
23999.459
32337.095
0.30 : 0.44
13731.054
24684.803
0.19 : 0.31
HCC-LM3
Bel-7402
8891.640
24778.459
0.11 : 0.34
10309.631
15076.388
0.14 : 0.19
